# RNA Viruses in Hymenopteran Pollinators: Evidence of Inter-Taxa Virus Transmission via Pollen and Potential Impact on Non-*Apis* Hymenopteran Species

**DOI:** 10.1371/journal.pone.0014357

**Published:** 2010-12-22

**Authors:** Rajwinder Singh, Abby L. Levitt, Edwin G. Rajotte, Edward C. Holmes, Nancy Ostiguy, Dennis vanEngelsdorp, W. Ian Lipkin, Claude W. dePamphilis, Amy L. Toth, Diana L. Cox-Foster

**Affiliations:** 1 Department of Entomology, The Pennsylvania State University, Pennsylvania, United States of America; 2 Department of Biology, Center for Infectious Disease Dynamics, The Pennsylvania State University, Pennsylvania, United States of America; 3 Mailman School of Public Health, Center for Infection and Immunity, Columbia University, New York, New York, United States of America; 4 Department of Biology, The Pennsylvania State University, Pennsylvania, United States of America; 5 Department of Entomology, University of Illinois, Urbana-Champaign, Illinois, United States of America; Institut Mediterrani d'Estudis Avançats (CSIC/UIB), Spain

## Abstract

Although overall pollinator populations have declined over the last couple of decades, the honey bee (*Apis mellifera*) malady, colony collapse disorder (CCD), has caused major concern in the agricultural community. Among honey bee pathogens, RNA viruses are emerging as a serious threat and are suspected as major contributors to CCD. Recent detection of these viral species in bumble bees suggests a possible wider environmental spread of these viruses with potential broader impact. It is therefore vital to study the ecology and epidemiology of these viruses in the hymenopteran pollinator community as a whole. We studied the viral distribution in honey bees, in their pollen loads, and in other non-*Apis* hymenopteran pollinators collected from flowering plants in Pennsylvania, New York, and Illinois in the United States. Viruses in the samples were detected using reverse transcriptase-PCR and confirmed by sequencing. For the first time, we report the molecular detection of picorna-like RNA viruses (deformed wing virus, sacbrood virus and black queen cell virus) in pollen pellets collected directly from forager bees. Pollen pellets from several uninfected forager bees were detected with virus, indicating that pollen itself may harbor viruses. The viruses in the pollen and honey stored in the hive were demonstrated to be infective, with the queen becoming infected and laying infected eggs after these virus-contaminated foods were given to virus-free colonies. These viruses were detected in eleven other non-*Apis* hymenopteran species, ranging from many solitary bees to bumble bees and wasps. This finding further expands the viral host range and implies a possible deeper impact on the health of our ecosystem. Phylogenetic analyses support that these viruses are disseminating freely among the pollinators via the flower pollen itself. Notably, in cases where honey bee apiaries affected by CCD harbored honey bees with Israeli Acute Paralysis virus (IAPV), nearby non-*Apis* hymenopteran pollinators also had IAPV, while those near apiaries without IAPV did not. In containment greenhouse experiments, IAPV moved from infected honey bees to bumble bees and from infected bumble bees to honey bees within a week, demonstrating that the viruses could be transmitted from one species to another. This study adds to our present understanding of virus epidemiology and may help explain bee disease patterns and pollinator population decline in general.

## Introduction

Pollinators of all types are vital to agriculture and are responsible for reproduction of crops worth >225 billion US dollars worldwide [1]. Honey bees (*Apis mellifera L.*) alone in the United States, account for an added market crop value exceeding 15 billion dollars [2]. However, pollinator populations in general have been declining over the last couple of decades [3]–[5]. The recent dramatic losses of thousands of honey bee colonies due to colony collapse disorder (CCD) and other causes [6], [7] has not only created great concerns in the scientific and agricultural community but has also highlighted the ever increasing risk of future crises in the global food supply due to our sole dependence on single pollinator species [8]. Apart from a pollination industry relying on only a few managed pollinators, more than 4000 other species of bees are native to North America. These non-*Apis* hymenopteran pollinators alone may be responsible for more than $3 billion of fruits and vegetables produced in US [9].

Being social insects, honey bees live in compact, highly organized and productive colonies consisting of up to 60,000 individuals. However, this social organization and the close interactions among colony members makes them highly susceptible to a variety of infectious diseases, among which viral pathogens are emerging as a serious threat to their health and survival [10], [11]. More than 18 viruses have been identified from different stages and castes of honey bees including eggs, larvae, pupae, adult workers, drones and queens from different parts of the globe [12]–[14]. Among these, four positive-sense, single-stranded RNA viruses are most common in the United States: Deformed wing virus (DWV), Black queen cell virus (BQCV), Sacbrood virus (SBV), and Kashmir bee virus (KBV) [10], [15]. Less commonly found are Acute bee paralysis virus (ABPV) and Chronic bee paralysis virus (CBPV). In addition, a recently described virus, Israeli acute paralysis virus (IAPV) was found to be highly associated with CCD-affected beekeeping operations throughout the US [16] and now appears to be more widely distributed nationwide [6]. IAPV sequence analysis across three genomic domains suggested the existence of three different groups: group 1 (the western strain) includes samples from operations in the western United States, as well as from bee packages imported from Australia; group 2 includes sequences from Israel; group 3 (the eastern strain) includes sequences from three operations in the eastern United States and one operation in Canada [17]. Elevated titers of multiple viruses were detected in bees from colonies with CCD symptoms [18]. All of these except CBPV have symmetric particles and a monopartite RNA genome. BQCV, ABPV, KBV and IAPV belong to a family Dicistroviridae, while DWV and SBV have been assigned to the genus *Iflavirus* [19], [20]. In contrast, CBPV has a multipartite genome organization and different particle morphology and has not yet been assigned to any genus or family [21].

Like most of the insect-infecting RNA viruses, so-called honey bee viruses usually persist as inapparent, asymptomatic infections, capable of replicating rapidly under certain conditions, resulting in observable symptoms often leading to colony losses [22], [23]. Symptoms of infections of the different viruses in honey bees range from deformed wings, discoloration, hair loss, bloated abdomens to trembling, paralysis, and brood and adult mortality, with serious consequences in terms of colony survival [10], [24]; the full impact on bee behavior and health by these different viral infections is not completely understood. A better understanding of the epidemiology of viruses is vital to understanding the dynamics underlying virus outbreaks and to shed light on the current honey bee and pollinator crises. Complex routes of virus transmission involving both horizontal as well as vertical transmission pathways have been documented in honey bees [10]. Transmission pathways include vector-borne transmission via *Varroa* mites (DWV [25], KBV [26], and recently IAPV [27]) and vertical transmission from infected queens and drones to their offspring [28]–[30]. In addition, detection of some of these viruses in glandular secretions of worker bees [31], in colony foods including pollen, honey and royal jelly [28], [29] as well as in bee feces [32], [33], suggests potential food-borne and fecal-oral routes of horizontal virus transmission inside the colony. Like other picornaviruses such as poliovirus [34], these viruses may infect a variety of tissues, with dissemination from the gut or site of infection affected by host conditions. The dissemination to other tissues and the impacts on bee health has not been extensively studied for these viruses [19].

Despite their designation as honey bee viruses, their host range is not restricted to *A. mellifera*, as there are some previous reports of these viruses from other pollinator species. Bailey and Gibbs [35] described ABPV as inapparent infection in bumble bee species. Later, KBV was detected in yellow jacket wasps (*Vespula germanica*) in Australia [36]. Recently, Genersch et al. [37] reported the occurrence of wing deformities in two bumble bee species (*Bombus terrestris and B. pascuorum*) in Europe, resembling those seen in DWV-infected honey bees. With molecular methods, they demonstrated that those bumble bees were indeed infected with DWV. A method has been recently published to detect ABPV, KBV, and DWV in bumble bees [38]. These reports suggest the possibility of wider environmental spread of these viruses with potential broader impact on the overall pollinator community.

Although, our understanding of viral epidemiology in honey bees has rapidly advanced over the last decade, most of the work has been focused on elucidating the routes of virus transmission within honey bee colonies. The intricate dynamics of interspecies virus transmission in the pollinator community has not been studied to date. Honey bees do not live in isolation in the environment, but mingle with other species on flowering plants. Other species include bumble bees, solitary bees, wasps, flies, ants, butterflies, mites and spiders, with which honey bees interact quite freely and frequently [39]–[42]. These interactions can lead to pathogen transmission; recent studies in bumble bees in Ontario, Canada have demonstrated pathogen spillover involving the spread of intestinal protozoa *Crithidia bombi* and *Nosema bombi* from commercial greenhouse bumble bees to wild native bumble bee species [43], [44]. The status of pollinators both in North America and Europe appears to be declining [4], [5]; how the decline of these essential members of our ecosystems relates to diseases is not known.

The focus of this study was to determine if pollen and/or pollinator species are involved in inter-taxa virus transmission in the pollinator community and to characterize the host range of RNA viruses. We have focused on viruses commonly associated with honey bees in the United States; DWV, BQCV, SBV and KBV, and the relatively newly-detected virus, IAPV. Viruses were detected using reverse transcriptase-PCR and confirmed by sequencing. In particular, we used phylogenetic analysis to study the distribution and sequence comparison of viruses in honey bee populations, the pollen loads collected by them from endemic, wild, flowering plants as well as agricultural crops, and other non-*Apis* pollinator species. We addressed several key questions: (i) Is the source of the RNA viruses in the stored pollen or bee bread potentially from the pollen forager or the pollen itself? (ii) How does the prevalence of viruses detected in pollen pellets compare to those found in foragers carrying those pellets? (iii) What is the association of the viruses with the pollen? (iv) Is the virus found in stored pollen infectious? (v) Are these viruses specific to honey bees or are they widespread in the hymenopteran pollinator community? (vi) Does phylogenetic analysis of viral sequences indicate interspecies viral transmission in the hymenopteran pollinator community? (vii) Can the transmission of viruses between honey bees and bumble bees be demonstrated experimentally?

## Results

### Is the source of the RNA viruses in the stored pollen or bee bread potentially from the pollen forager or the pollen itself?

**Figure 1:**
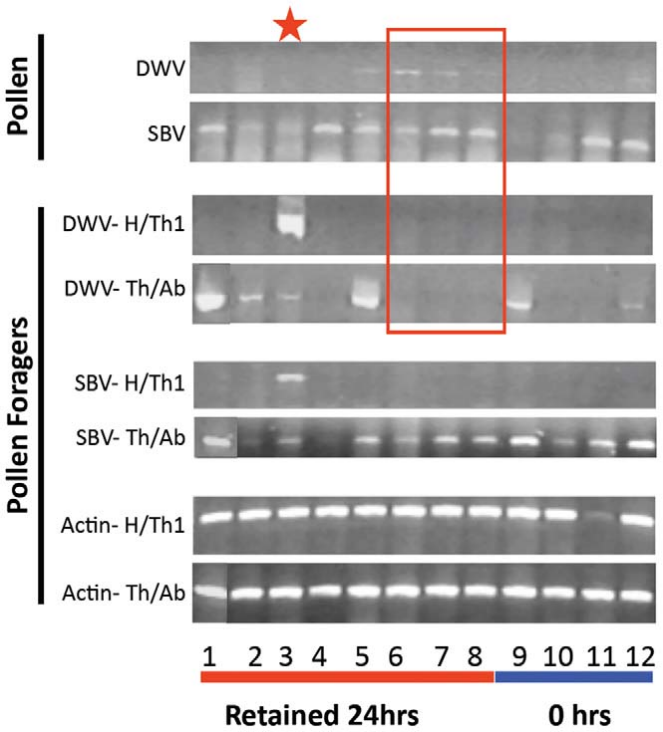
Comparison of viral presence in pollen pellets and their corresponding forager with her body dissected into two regions with or without salivary glands, to determine if salivary secretions of the forager are associated with virus in pollen pellets. Incoming foragers with pollen pellets were collected in 2005; pollen pellets removed, tagged with identifier, and frozen at −80°C. Some foragers (1–8) were kept for 24 hrs at 34°C, 50% relative humidity and fed sugar water; others (9–12) were frozen immediately upon collection. After freezing, all foragers were divided into two regions, head plus prothorax that have salivary glands **(H/T1)** and the remainder of body lacking salivary glands **(T2,3/A)**. Pollen pellets and forager body regions were extracted for detection of deformed wing virus (DWV), sacbrood virus (SBV), and actin mRNA (forager only). Actin mRNA was used as an internal control for methods and loading. Red box indicates three foragers that lack detections of DWV but had pollen pellets with detectable DWV. The red star (lane 3) indicates a forager with heavy DWV infection in Head/Prothorax but no detectable DWV in her pollen pellets. Size of DWV reaction = 424 bp, SBV reaction = 693 bp and Actin reaction = 514 bp.

Of the 12 initial honey bee pollen foragers analyzed for SBV and DWV and either kept for 24 hrs after removing their pollen pellets or directly frozen, there were no detectable differences in the prevalence of the viruses in these two groups. This suggests that the supply of virus associated with either the salivary glands or digestive tract was not reduced in those assayed immediately after collection as compared to those kept for 24 hrs. All foragers were dissected into two regions prior to virus analysis, the head and first thoracic segment containing the salivary glands and the remainder of the body. Three of the foragers were found to have detectable DWV in their pollen loads, without any detectable viral infections in the head/thoracic segments and two of these did not have any detectable infections anywhere (Figure 1) indicating that the salivary secretions of the forager were not a likely source of viral contamination on the pollen. One forager had a high level of DWV in her head/thoracic segment but there was no detectable DWV in her pollen load. SBV was detected in most of the pollen pellets as well as in the abdomen/thorax of the foragers, but few of the foragers had detectable virus in their heads/thoracic segments. These data therefore suggest that there is an alternative source of bee bread contamination.

### How does the prevalence of viruses detected in pollen pellets compare to those found in foragers carrying those pellets?

**Figure 2:**
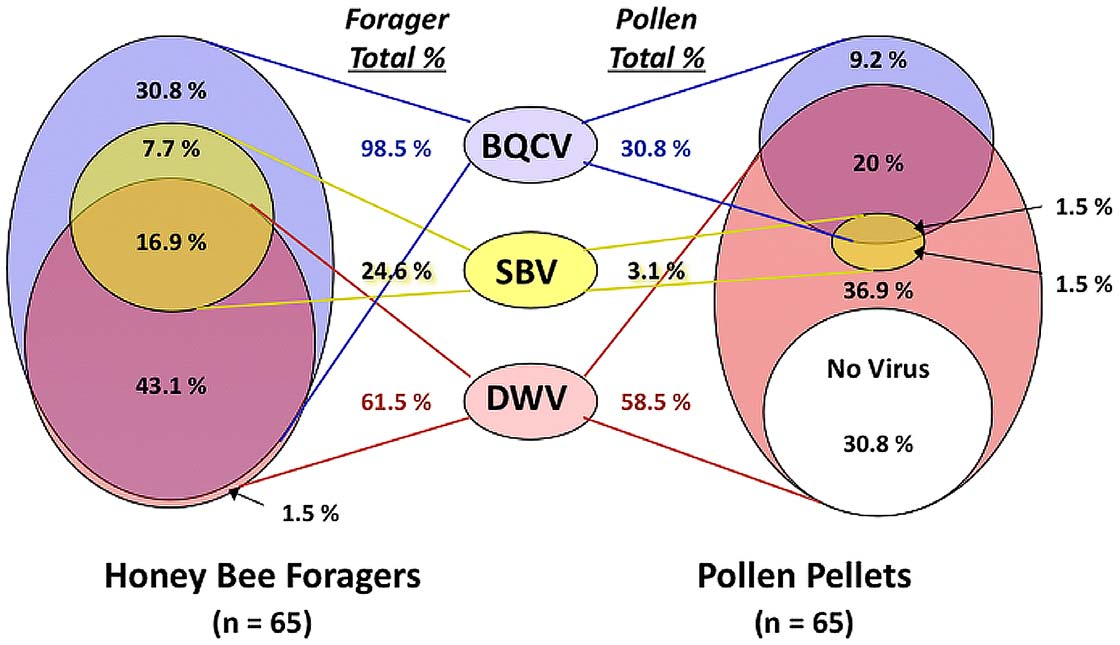
Proportion of virus species detected in 65 honey bee pollen foragers versus their pollen pellets. For both the foragers and their pollen pellets, Venn diagrams depict the percentage of DWV (Deformed wing virus in red), SBV (Sacbrood virus in yellow), BQCV (Black queen cell virus in blue), or virus-free samples (white). Overlapping colored circles indicate samples wherein more than one virus was detected. Total percentages of these viruses in either foragers or pollen pellets are given in the middle of the figure. N = sample size.

In an expanded collection of 65 honey bee pollen foragers in 2007, all foragers were infected with at least one virus with most having multiple infections. A large number of pollen pellets also were positive for one or more virus species. BQCV was the most prevalent species detected in the honey bee samples (98.5%). In comparison, only 30.8% pollen pellets were positive for this virus (Figure 2, Table 1). SBV was less common with only 24.6% bees and 3.1% pollen pellets detected positive. The incidence of DWV was relatively high, and this virus was almost equally detected among foragers (61.5%) and their pollen loads (58.5%). All forager honey bees and their pollen loads tested negative for IAPV and KBV. DWV was the most commonly detected virus in pollen loads of honey bees. More importantly, there were forager/pollen pellet pairs where the uninfected forager was carrying pollen loads positive either for DWV (13.9%) or SBV (1.5%) (Table 1, Figure 2).

**Table 1:** Prevalence of RNA viruses in honey bee foragers and their corresponding pollen pellets collected from multiple hives and apiaries in central Pennsylvania from June to September 2007.

VIRUS		% Infected	% Non-Infected	Total %
DWV		Foragers	Foragers	Pollen Pellets
	**% Pollen Pellets**	44.6	13.9	58.5
	**(+) Virus**	(29)*	(9)	(38)
	**% Pollen Pellets**	16.9	24.6	41.5
	**(−) Virus**	(11)	(16)	(27)
	**Total % Foragers**	61.5	38.5	
		(40)	(25)	

		% Infected	% Non-Infected	Total %
SBV		Foragers	Foragers	Pollen Pellets
	**% Pollen Pellets**	1.6	1.5	3.1
	**(** + **) Virus**	(1)	(1)	(2)
	**% Pollen Pellets**	23.0	73.9	96.9
	**(** − **) Virus**	(15)	(48)	(63)
	**Total % Foragers**	24.6	75.4	
		(16)	(49)	

		% Infected	% Non-Infected	Total %
BQCV		Foragers	Foragers	Pollen Pellets
	**% Pollen Pellets**	30.8	0	30.8
	**(** + **) Virus**	(20)	(0)	(20)
	**% Pollen Pellets**	67.7	1.5	69.2
	**(** − **) Virus**	(44)	(1)	(45)
	**Total % Foragers**	98.5	1.5	
		(64)	(1)	

DWV: Deformed wing virus.

SBV: Sacbrood virus.

BQCV: Black queen cell virus.

*Number of individual foragers and their pollen pellets in category are indicated in parentheses.

+Positive for virus.

−Negative for virus.

Overall, there was a significant, positive association between the prevalence of virus species found in the forager and in the pollen pellet of that forager (Cochran-Mantel Haenszel Statistic  = 9.46; df = 1; p = 0.002). However, this relationship seemed to be driven mainly by DWV frequencies. Analyzing frequencies of the three viruses separately revealed a significant correlation between the prevalence of DWV positive foragers and the DWV positive pollen pellets (Fisher Exact test; p = 0.005). This relationship between forager and pollen also extended to the other pollinator species collected at the same time; among three pollen pellets taken from non-*Apis* hymenopteran pollinators, one was positive for DWV (data not shown) and the corresponding forager bumble bee was also positive for DWV. However, there was no significant, positive correlation between the frequencies of foragers and pollen pellets with SBV (Fisher Exact test; p = 0.435) or BQCV (Fisher Exact test; p>0.999). There were significantly fewer samples where both forager and corresponding pollen pellet were positive for SBV or BQCV in comparison to the pairs where only the forager was positive while her corresponding pollen pellet was negative (Table 1).

The frequency of the number of co-existing virus species was significantly different between the honey bee foragers and the pollen pellets (Mantel-Haenszel χ^2^ = 36.36; p<0.001). A higher percentage of bees had two or three viruses co-existing versus only one virus; however, the trend was opposite in pollen (Figure 2). All the honey bees were infected with at least one virus; in comparison, 30.8% pollen pellets were free from any virus tested in this study. Co-infections of three viruses (DWV, SBV and BQCV) were detected in 16.9% of honey bee foragers; while, only 1.5% pollen pellets tested positive for all three viruses.

### What is the association of the viruses with the pollen?

Based upon color of pollen pellets (Figure S1) and pollen morphology, the virus-positive pollen belonged to many plant species including goldenrods (*Solidago* spp.), thistles (*Cirsium* spp.), clovers (*Trifolium & Melilotus* spp.) and common burdock (*Arctium minus*).

**Figure 3:**
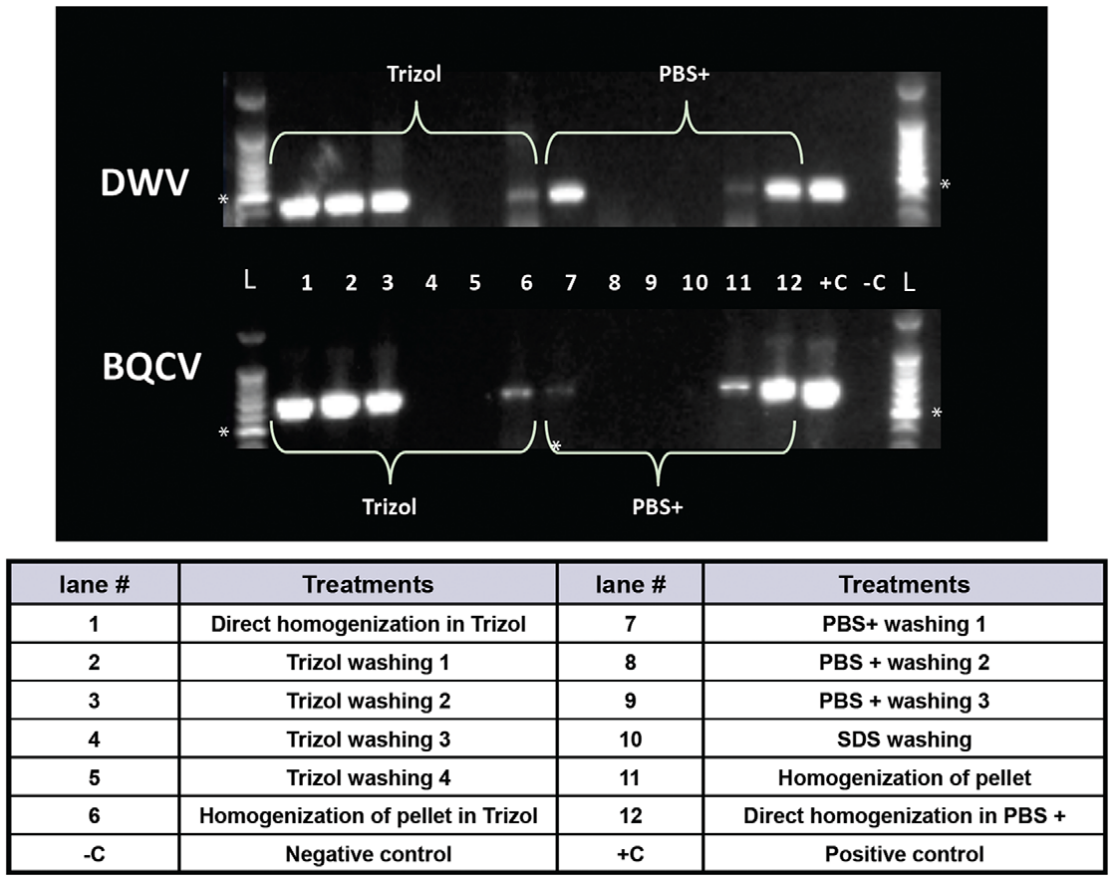
Determination of how DWV and BQCV are associated with pollen. Virus was detected in extracts of supernatants and homogenates after various washings of an aliquot of combined pollen pellets, in either Trizol or PBS+ (1M PBS, 0.05% Tween, 2% Polyvinylpyrrolodone) followed by SDS (Sodium Dodecyl Sulfate). Standard size ladders (L) are shown at beginning and end of gel images. Size marker of 500 bp is indicated by * on the ladder. Lane loadings are indicated below the gel image. Size of DWV reaction = 424 bp and BQCV reaction  = 700 bp.

The pollen-virus association was further examined to determine if there was potential for the viruses to be inside the pollen grain as opposed to just being on the outside of the pollen exine. Following extensive rinses with Trizol, both DWV and BQCV were present in the supernatants from first two washings. However, no virus was detected in the third and fourth washings but was again detected in the homogenized pollen, although the viral RT-PCR bands on the gel were less intense as compared to the bands in the first two washings (Figure 3). Similar results were obtained with phosphate saline-Tween polyvinylpyrrolodone buffer (PBS+) and sodium dodecyl sulfate (SDS) washings. This suggests a possibility for a more intimate association between these RNA viruses and plant-pollen.

### Is the virus found in stored pollen infectious?

**Figure 4:**
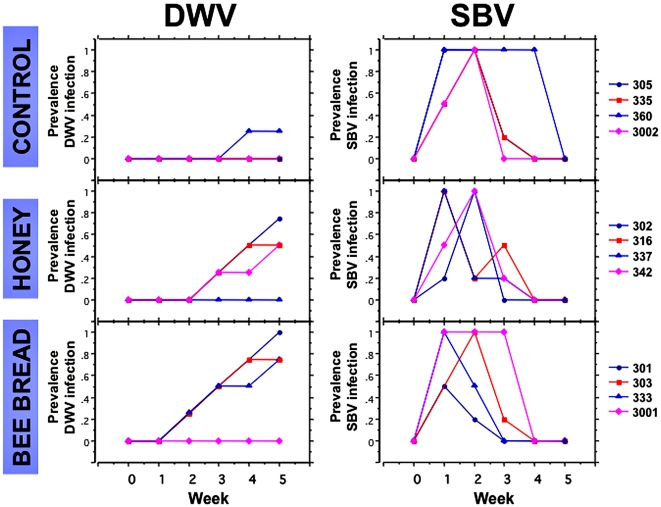
Test of DWV infectivity in stored pollen and honey and detection of SBV movement from outside source through transmission of DWV and SBV to eggs by queens. DWV- and SBV-free colonies were installed into new equipment in an isolated apiary near State College, Pennsylvania in the spring of 2005. Colonies in the **Control** treatment were each fed sugar water and artificial pollen, plus given a washed frame. In the **Bee Bread** or stored pollen treatment, colonies were each given a frame of bee bread with detectable DWV and sugar water. In the **Honey** treatment, colonies were each given a frame of capped honey with detectable DWV and artificial pollen diet. No SBV was detected in the workers or eggs from the queens in the colonies or the frames of honey or stored pollen prior to experiment. Egg samples (4 samples of 5 eggs each; 20 eggs total per colony) were collected weekly from each of four colonies (colony numbers listed on right of figure) in three treatment groups, at time of feeding and for five additional weeks. Eggs were extracted and used for detection of DWV, SBV and actin mRNA (present in 100% samples, not shown). Detection of actin mRNA in the honey bees is used as an internal control for extraction efficiency.

A field experiment demonstrated that viruses detected in bee bread (stored pollen) and honey were infectious. In an apiary isolated from other known honey bee colonies, DWV-free colonies were given either bee bread with DWV, honey with DWV, or virus-free artificial foods (control). To avoid false positives originating from the detection of virus in the gut of the honey bees that had consumed the virus-contaminated bee bread (stored pollen), virus infection was monitored in eggs laid by the queen. For the queen to become infected and to lay infected eggs, workers that attend to the queen and feed her royal jelly from their salivary glands would need to become infected and actively secrete virus in the royal jelly. Any virus infection in the colony workers would have to be via the consumption of the virus-contaminated foods. By the end of week 2, the bee bread was entirely consumed by the workers in all four colonies in the treatment; the frames of honey were consumed by the end of week 3 (Figure 4).

DWV was not detected in egg samples from all 12 colonies for the first week following introduction of the frames of virus-contaminated bee-bread, honey, or “clean” frames (Figure 4). At week two, three out of four colonies fed virus-contaminated bee-bread were found to have queens laying eggs with detectable DWV; in subsequent weeks, the percentage of the egg samples infected with the virus increased in these three colonies. With a delay of one week, a similar pattern was observed in colonies fed with DWV-contaminated honey, with three out of four colonies having eggs positive for DWV. Only one control colony had a few DWV infected eggs by weeks four and five. The percentage of colonies infected with DWV over time was significantly higher in treatments where either contaminated bee bread or honey was fed as compared with controls (two-way ANOVA, treatment p<0.0001, time p<0.001; Tukey-Kramer post hoc analysis p<0.05 for treatments). This indicated that DWV virus in the stored pollen or honey was infectious, even after storage in a pest-free building at ambient outdoor temperature (fluctuating from approximately -6°C to 32°C) for six months.

In this experiment, SBV was also monitored in the same samples and from the same cDNAs tested for DWV, as described above. Although initially all colonies and the foods fed to the bees were SBV-free, all 12 colonies had eggs positive for SBV beginning in week 1 (Figure 4), with no significant differences in SBV prevalence among the treatments (ANOVA, p = 0.84). This suggested that SBV came into the colonies from an outside source. However, by the end of week 5, SBV was no longer detectable in any of the egg samples from the 12 colonies. Interestingly, these samples with declining prevalence of SBV were eggs in which the DWV prevalence was increasing (Figure 4). The disappearance of SBV but continued infection by DWV suggested that the queen could selectively clear infection by one virus species while continuing to be infected by another related virus.

### Are these viruses specific to honey bees or are they widespread in the hymenopteran pollinator community?

Eleven non-*Apis* hymenopteran species, collected from flowering plants near the honey bee apiaries, were positive for one or more virus species (DWV, BQCV, SBV, KBV, IAPV) (Table 2). These included three common bumble bee species (*Bombus impatiens*, *B. vagans, B. ternarius*), the eastern carpenter bee (*Xylocopa virginica*), the small carpenter bee (*Ceratina dupla*), a sweat bee (*Augochlora pura*), mining bees (*Andrena* sp.), a yellow jacket (*Vespula vulgaris*), paper wasps (*Polistes metricus, P. fuscatus*) and sand wasp (*Bembix* sp.). Moreover, IAPV was detected only in non-*Apis* hymenopteran pollinators collected near the apiaries harboring honey bees with IAPV, from Pennsylvania and New York.

**Table 2:** RNA viruses detected in non-*Apis* hymenopteran pollinators collected from flowering plants in Pennsylvania, New York and Illinois from May to October 2007.

Collected near IAPV(-) honey bee apiaries in PA						
Pollinator Species	IAPV	DWV	BQCV	SBV	KBV	Co-Infections*
*Andrena* sp.	−	+	−	−	−	
Mining bees (n = 4)		(2)				
*Bembix* sp.	−	+	+	−	−	DWV+BQCV
Sand wasps (n = 2)		(1)	(1)			
*Bombus impatiens*	−	+	+	−	−	DWV+BQCV
Eastern bumble bee (*n* = 5)		(5)	(3)			
*Bombus* sp.	−	+	+	+	+	DWV+BQCV+ SBV+KBV
Bumble bees (*n* = 3)		(3)	(1)	(3)	(3)	
*Ceratina dupla*	−	+	−	−	−	
Small carpenter bee (n = 1)		(1)				
*Vespula vulgaris*	−	+	+	−	−	DWV+BQCV
Yellowjacket wasp (n = 5)		(4)	(2)			
*Xylocopa virginica*	−	+	+	−	−	DWV+BQCV
Eastern carpenter bee (*n* = 4)		(3)	(2)			

Collected near IAPV(+) honey bee apiaries in PA and NY						
Pollinator Species	IAPV	DWV	BQCV	SBV	KBV	Co-Infections*
*Andrena* sp.	−	+	+	+	−	DWV+BQCV+SBV
Mining bees (n = 4)		(3)	(1)	(1)		
*Augochlora pura*	+	+	−	−	−	IAPV+DWV
Sweat bee (n = 1)	(1)	(1)				
*Bombus ternarius*	+	+	+	+	−	IAPV+DWV+SBV
Tricolored bumble bee (n = 2)	(2)	2)	(1)	(1)		
*Bombus vagans*	+	+	+	+	−	IAPV+DWV+SBV+BQCV
Bumble bee (n = 1)	(1)	(1)	(1)	(1)		
*Ceratina dupla*	−	+	−	−	−	
Small carpenter bee (*n* = 2)		(1)				
*Polistes fuscatus*	−	+	−	−	−	
Paper wasp (n = 6)		(1)				
*Vespula vulgaris*	+	+	+	+	−	IAPV+DWV+SBV+BQCV
Yellowjacket wasp (*n* = 7)	(5)	(7)	(6)	(4)		

Collected in IL						
Pollinator Species	IAPV	DWV	BQCV	SBV	KBV	Co-Infections*
*Polistes metricus*	−	+	+	+	−	DWV+SBV or SBV+BQCV
Paper wasp (*n* = 8)		(4)	(2)	(4)		

IAPV: Israeli acute paralysis virus; DWV: Deformed wing virus.

SBV: Sacbrood virus; BQCV: Black queen cell virus.

KBV: Kashmir bee virus; PA: state of Pennsylvania, USA.

NY: state of New York, USA; IL: state of Illinois, USA.

CCD: Colony collapse disorder; n =  Total number of individuals tested.

−Negative for virus;

+Positive for virus, (# of samples with virus).

*Represented by the individual detected with maximum number of co-infecting viruses.

### Does phylogenetic analysis of viral sequences indicate interspecies viral transmission in the hymenopteran pollinator community?

A comparison of viral sequences from the honey bee foragers, their pollen pellets and from the non-*Apis* hymenopteran pollinators revealed the relationship among viral strains and pollinator species as well as the relationship between each forager and pollen pellet combination. The analysis of samples taken near IAPV-free apiaries was restricted to BQCV and DWV, given the lack of significant number of samples positive for SBV. The IAPV sequences from the non-*Apis* hymenopteran pollinators were also compared to the sequences obtained from honey bees taken from apiaries with IAPV and a known history of CCD symptoms.

**Figure 5:**
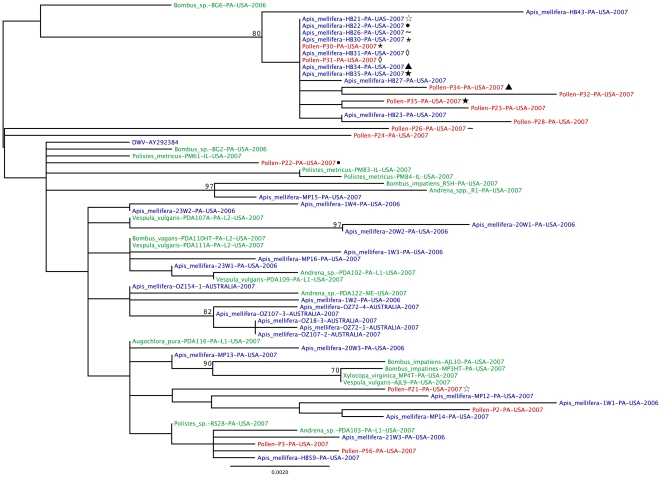
Phylogenetic comparison of DWV sequences detected in honey bee foragers, pollen pellets, and non-*Apis* hymenopteran pollinators. An unrooted maximum likelihood phylogenetic tree of DWV (based on 1230-nt from the capsid) was generated using a region of the structural proteins of the virus. The support for the indicated branching topology was evaluated by using bootstrap re-sampling of the sequences 1,000 times. Nodes supported by bootstrap values over 70 are given. Strains are annotated by genus, species, identification-label, country of isolation, and year of isolation. Blue =  virus from honey bee, red =  virus from pollen pellet, and green =  virus from non-*Apis* hymenopteran pollinators. Forager/pollen pellet pairs are indicated by common symbols following the sample label.

**Figure 6:**
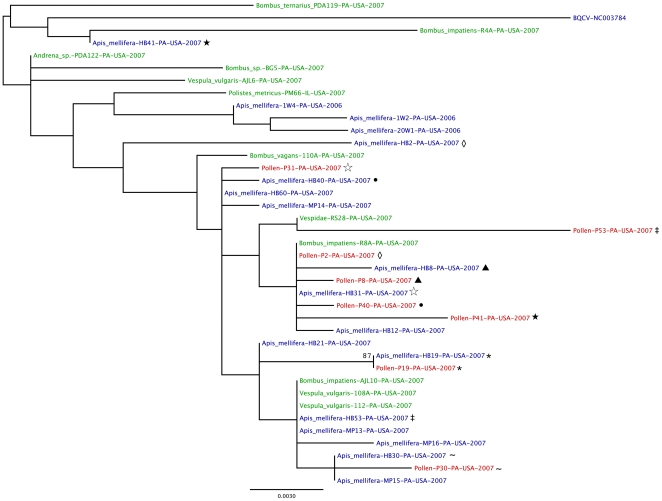
Phylogenetic comparison of BQCV sequences detected in honey bee foragers, pollen pellets, and non-*Apis* hymenopteran pollinators. An unrooted maximum likelihood phylogenetic tree of BQCV (based on 687-nt from Capsid/3′UTR) was generated using a region of the structural proteins of the virus. The support for the indicated branching topology was evaluated by using bootstrap re-sampling of the sequences 1,000 times. Nodes supported by bootstrap values over 70 are given. Strains were annotated by genus, species, identification label, country of isolation and year of isolation. Blue =  virus from honey bee, red =  virus from pollen pellet, and green =  virus from non-*Apis* hymenopteran pollinators. Forager/pollen pellet pairs are indicated by common symbols following the sample label.

Maximum likelihood (ML) phylogenetic trees were inferred for DWV (Figure 5) and BQCV (Figure 6) using a region of genes (see Table S1 for exact location) encoding the structural capsid proteins in the polyprotein for each of these viruses detected in the *Apis mellifera* foragers, their pollen pellets, and other non-*Apis* pollinators. The strength of species-specific association of the viruses was then assessed statistically using the BaTS (Bayesian tip-association significance testing) program. For both DWV and BQCV, there was no obvious clustering of viral sequences by the type of pollinator from which it was taken (Figures 5 & 6); instead, the sequences were intermixed across the trees. Indeed, there was no significant signal for clustering of the virus by host species in the BQCV phylogenetic tree [Association Index (AI), *P* = 0.167; Parsimony Score (PS), *P* = 0.119], such that BQCV strains are no more associated with specific pollinator species than random. DWV, however, had a weakly significant phylogenetic separation by species (AI, *P* = 0.023; PS, *P* = 0.02). This significance in DWV clustering, however, is strongly confounded by collection dates (AI, *P* = 0; PS, *P* = 0), as opposed to an actual species-specific difference. This in part reflects the difference in collection dates for the forager honey bees and the other pollinators, and further suggests that any species-specific clustering in DWV is very weak.

In addition, there was no obvious clustering of sequences of the virus found in the pollen pellets separately from the viral sequences from the pollinators (Figures 5 & 6). This suggests that the virus found in pollen was most likely previously deposited on the flowers by pollinators infected with the virus.

**Figure 7:**
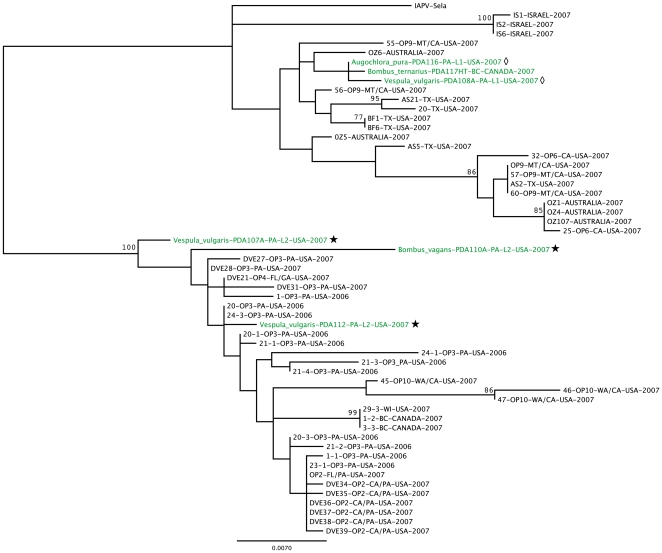
Phylogenetic comparison of IAPV sequences detected in honeybees and non-*Apis* hymenopteran pollinators collected near IAPV(+) apiaries. An unrooted maximum likelihood phylogenetic tree of IAPV (based on 771-nt for capsid region) was generated using a region of the structural proteins of the virus. The support for the indicated branching topology was evaluated by using bootstrap re-sampling of the sequences 1,000 times. Nodes supported by bootstrap values over 70 are given. Strains were annotated by genus, species, identification label, country of isolation and year of isolation. Green =  virus from non-*Apis* hymenopteran pollinators; Black  =  virus sequences from original isolation and honey bees from CCD-affected operations [16]. Non-*Apis* hymenopteran pollinators collected from same local are indicated by common symbol following sample label.

Similarly, for IAPV, an ML phylogenetic tree was inferred using a part of the structural polyprotein for the virus from the *Apis mellifera* specimens taken from apiaries diagnosed with CCD [16], [17] and the non-*Apis* pollinators collected from near two of the IAPV-infected apiaries (associated with Operation 3 [16]). The IAPV detected in the non-*Apis* pollinators did not cluster separately from the IAPV in the honey bees in nearby apiaries (Figure 7) (AI, *P* = 0.083; PS, *P* = 1). Interestingly, the viral sequences from non-*Apis* hymenopteran pollinators collected from the two apiaries segregated by sampling location, with the non-*Apis* hymenopteran pollinators from each site possessing a clearly phylogenetically distinct lineage of IAPV.

### Can the transmission of viruses between honey bees and bumble bees be demonstrated experimentally?

In experimental greenhouse rooms that housed both bumble bees and honey bees along with flowering plants, bumble bees were observed to visit the flowering plants along with honey bees, foraging at the same time. No bumble bees were observed to collect powdered Megabee artificial pollen diet that was provided for honey bees and rarely did bumble bees collect sugar water at the common feeders.

IAPV was used as a test virus for interspecies transmission, given that IAPV-free honey bee colonies as well as purchased bumble bee colonies could be obtained. In the 2008 experiment, one week after feeding IAPV in sugar solution to honey bee colonies, workers from those colonies had detectable IAPV, while worker bees from colonies fed only sugar water in the control room were remained virus free. In the three bumble bee colonies co-existing and co-foraging in the rooms with the IAPV-infected honey bees, bumble bee workers from one of the colonies tested positive for IAPV on week two onwards; while, no IAPV was detected in any of the worker bumble bees from the three colonies in the IAPV-free room.

In a repeat of the experiment in 2009, bumble bees from the same supplier used in the previous year arrived already infected with the western strain of IAPV. The movement of different strains of IAPV between honey bees and bumble bees was successfully tracked. After keeping IAPV-free honey bees with infected bumble bees in the same room for 10 days, the western strain of IAPV was detected in one of the three honey bee hives. When honey bees were fed with the eastern strain of IAPV, this eastern strain moved into all three bumble bee hives (8 out of nine bumble bees tested positive) within a week; whereas, none of the bumble bee hives in the control room (without eastern strain of IAPV being fed) tested positive for the eastern strain of IAPV. These results demonstrated that IAPV could move easily between honey bees and bumble bees with the only contact being common visits to flowers.

## Discussion

### Is the forager transferring the virus into the pollen pellet that she carries?

We report in this paper the first molecular detection of DWV, BQCV and SBV in the pollen loads directly taken from the pollen baskets of forager honey bees. A high percentage of tested pollen loads were positive for these viruses, especially for DWV. Similarly, one of three analyzed pollen pellets from non-*Apis* hymenopteran pollinators had DWV. These data suggest that pollen can be frequently associated with RNA viruses that infect bees and that that the virus detected in the bee bread or pollen stores in the hive is in part due to virus associated with the pollen itself.

Overall, there was significant variation in the distribution of different virus species in foragers and their corresponding pollen loads. While BQCV was the most prevalent virus in the honey bee foragers, comparatively fewer pollen loads carried the virus. Conversely, the incidence of DWV was equally prevalent in foragers and their pollen loads. These differences in prevalence rates suggested that the different viruses differ in their viral ecology in this environment, either in different infection rates among the pollinators or in their transfer to the pollen. Given that one third of all pollen pellets from honey bee pollen foragers were free of detectable virus in spite of all foragers being infected, these viruses may not be frequently transferred to the pollen loads by pollen foragers. Instead, a different group of pollinators may be responsible for contamination of pollen. DWV was in fact the most prevalent virus in the wild pollinators, which may help explain the increased detection of DWV in pollen pellets of honey bee foragers. The viral ecology and infection dynamics in pollinators demand further study.

The high prevalence of BQCV and DWV in honey bees in our study was consistent with results from other virus surveys in the US [10], [15]. Notably, multiple virus species were found co-infecting non-*Apis* hymenopteran pollinators as well as honey bees in this study, which corroborate many reports of multiple viral co-infections in honey bees throughout the world [15], [18], [45], [46]. This is especially important since multiple viruses have been found associated with CCD, without one viral agent or other pathogen being linked to CCD by itself [46]–[48]. In addition, multiple viruses were detected in the pollen loads, although the percentage of pollen loads with multiple virus species was significantly less compared to the foragers.

Perhaps the most important observation was the detection of DWV and SBV in pollen loads of uninfected foragers suggesting that some foragers were bringing in virus from outside and thereby directly implicating pollen as a source of virus infection for healthy colonies. We did not find any uninfected foragers bringing in BQCV in pollen loads, since the incidence of this virus was very high in foragers (almost 100%). Previous research on plant viruses demonstrated that, by moving pollen from plant to plant, honey bees play an important role in the transmission of some pollen-borne plant viruses [49], [50], which complements our findings. The implication of our finding is that pollinators may become infected with a new virus introduced to the environment by another species, mediated by plant pollen.

How the virus becomes associated with the pollen is not known. Historically, it was assumed that the forager collects the pollen and, through the addition of salivary secretions to moisten the pollen, she molds the pollen into a pellet to pack it into her pollen basket [51]. Previous studies have reported the detection of some of these RNA virus species in the thoracic and hypopharyngeal salivary glands of honey bees [24], [31]. This is further supported by detection of several of these viruses in the colony foods including honey, pollen and royal jelly [28], [29]. Our data indicate it is unlikely that the salivary secretions of the foragers transfer the virus to the pollen given three findings: 1) pollen pellets can be found to have virus without the forager herself having detectable virus, 2) not all the pollen pellets from infected foragers had virus, and 3) only a small fraction of the viral sequences matched between a forager and her pollen pellet, in the pairs with detectable virus.

Alternatively, pollen may be contaminated with virus via other means. One of the potential routes is via random deposition of feces from infected insects on flowers. This alternative is supported by reports of detection of several viruses in the honey bee feces- CBPV [52], KBV [33], DWV & BQCV [32] and most recently IAPV (Singh et al, unpublished). Honey bees and bumble bees are known to defecate in the field while foraging. The role of the digestive tract in virus transmission is supported by detection of significantly higher virus titers in the digestive tracts of honey bees as compared to other tissues [32]. Also the infectivity of virus from bee feces has been proven, both by injecting healthy bees with virus particles obtained from feces of infected bees and more importantly, by keeping naive bees in the feces-soiled environment [53]. Most studies have implicated virus in the fecal matter as one of the routes of horizontal transmission in the honey bee hives, but this could also be the mechanism underlying the inter- and intra-species virus transmission via pollen.

It is unknown how long these ssRNA viruses can survive on flowers under harsh environmental conditions including UV radiation, high temperature and desiccation. On the other hand, for inter-taxa virus transmission to take place, viruses may not need to survive for long periods, as the interactions between pollinators on flowers can be quite intense and frequent, especially during full bloom.

### What is the nature of the association of viruses with the pollen itself?

The pollen that tested positive for the virus was not associated with any particular plant species. Rather, the virus-positive pollen pellets belonged to many plant species including goldenrods, thistles, clovers and common burdock. These pollen sources represent a diverse group, suggesting no restriction by the plant taxonomy on the viral association with the plant. Determining the diversity of plants and the timing of the association with the pollen is important in understanding if the plant plays any role in this viral transmission route in the pollinator community.

Potentially some of the plants may be serving as the reservoirs of these viruses. Detection of viral RNA in the supernatant from the first two pollen washings (washings with Trizol or PBS and SDS solutions) and then pollen homogenate after a fourth washing suggests that either the virus particles were present both on the surface of pollen grains as well as inside the pollen grains or that they were tightly bound to the pollen exine. For pollen-vectored plant viruses, the virions can be located both inside and outside the pollen grains [54], [55]. The order Picornavirales, to which these picorna-like viruses belong, is known to contain viruses infecting plants and animals, including humans [56], [57]. Recently these viruses were found to have different patterns of dinucleotide bias dependent upon their host (insect, plant, or mammal) [58]; although, some of the insect and plant viruses did not cleanly differ in their dinucleotide bias, suggesting that there is potential for some viruses to infect both insects and plants. A dicistrovirus that infects aphids can also become associated with and persist in plant phloem cells [59].

### Is the virus associated with pollen or honey infective?

The viruses detected in the food stores of the honey bees were found to be infective even after being kept at ambient temperature for several months. In the bee bread, the pollen pellets are packed into separate layers, with different pollen sources being found in differently colored layers. Different viruses can be detected in different layers (data not shown), indicating that viruses associated with each layer were present in the forager-collected pollen pellets.

Despite the environmental exposure of the virus to sun and desiccation, these viruses remained infective when fed to honey bee colonies. Environmental stability after exposure to sunlight, desiccation, temperature fluctuation, and microbial degradation has been reported for other picornaviruses, such as poliovirus in polluted water and for foot and mouth virus transmitted by wind currents [60]–[62]. Pollen-borne plant viruses vectored by honey bees during pollination are known to remain infective for several weeks after being stored in the bee bread in the hive [63]. It is not known what the infective period is for the picornaviruses detected in pollen and honey, especially under different conditions. This would be valuable information for beekeepers and aide in understanding viral disease dynamics.

### Are these viruses specific to honey bees or are they widespread in non-*Apis* hymenopteran pollinators?

Knowledge of the degree of host specificity is important to the understanding of pathogen transmission dynamics. Detection of one or more RNA viruses from as many as 11 non-*Apis* hymenopteran species demonstrated that these picornaviruses are widely distributed in the pollinators and are not specific to honey bees or their close relatives, given that each of these viral-species effectively represents a single genetic population. Understanding disease dynamics and tracking outbreaks requires broadening consideration to the community level instead of solely focusing on individual host-pathogen interactions.

For DWV and BQCV, our data indicate that there are not distinct segregations of the viral populations among the pollinators in the temporal and spatial confines of the study. Even when the DWV and BQCV sequences from *Polistes* wasps in Illinois are compared to the viral sequences from pollinators and pollen in Pennsylvania, there is no significant segregation. This suggests that the same viral strains are circulating amongst these diverse species. For other viral diseases in animals, this lack of segregation in phylogenetic analysis of viral sequences taken from different host species has indicated that the cellular mechanisms regulating infection are not highly constrained among these hosts [64]. The honey bees and non-*Apis* hymenopteran pollinators may have similar viral receptors, permitting each to become infected with these picorna-like viruses. This is not common among all insects, since restrictive host range for other dicistroviruses has been observed in widely separate insect taxa [65].

Importantly, IAPV was detected only in non-*Apis* hymenopteran pollinators collected near IAPV-infected apiaries in New York and Pennsylvania. None of the non-*Apis* hymenopteran pollinators collected from flowering plants around State College, Pennsylvania, where IAPV was not detected in 2007 in honey bees, tested positive for this virus. IAPV may be spreading into non-*Apis* hymenopteran pollinators from honey bees. Alternatively, this virus may have spilled over from some wild species, which may be serving as the reservoir host for this virus, into honey bees. Moreover, results from our greenhouse experiment on interspecies transmission also indicate that this virus does not have any directionality at least between honey bees and bumble bees in a controlled environment.

### Does pollen play any role in inter-taxa transmission?

The role of pollen in interspecies virus transmission is supported by phylogenetic analysis. Since there was no clustering of any virus according to whether it was isolated from honey bees, wild pollinators or from pollen, it is highly likely that the same viral strains are freely circulating in the pollinator community and that pollen serves as a mediator of viral transmission. The predominant mismatch in sequences of viruses in the forager/pollen pairs also strongly suggests that most foragers were carrying pollen loads with virus originating from other individuals.

Importantly, pollen can have a role in virus transmission among pollinators without the reservoir-host species being in the same locale. In our greenhouse study on the interspecies transmission of viruses between honey bees and bumble bees, we obtained bumble bee colonies from two different vendors in two different years. During the first year trials, tests of bees from one vendor revealed that all six bumble bee colonies came already infected with IAPV, while the colonies from the other vendor were IAPV free. In replicate trials the next year, colonies purchased from the previous year's IAPV-free vendor arrived infected with IAPV. Both companies reported that honey bee-collected pollen purchased from honey bee operations in the US and Canada was used in rearing the bumble bees. We surmise that this pollen was contaminated with IAPV and served as the vehicle for viral transmission into these colonies. More than 200 tons of honeybee-collected and preferably freshly-frozen pollen is used annually for bumble bee rearing worldwide [66]. This same concern may also extend over to honey bees, since many beekeepers purchase pollen to feed their bees. Pollen has been successfully gamma irradiated without destroying nutritional and physical properties [67], and this practice should be encouraged to prevent introduction of new strains of viruses and other pathogens, irrespective of the source of the pollen.

In conclusion, we propose that pollen serves as one of the major routes of inter-taxa virus transmission in the pollinator community. This is supported by Bailey's report [51] of the presence of SBV and CBPV in the pollen loads of honey bees and presence of ABPV in pollen loads of both honey bees and bumble bees. The dynamics of this viral transmission route via pollen need to be further defined to understand how the multiple viruses move from one species to another, and to determine if pollen and its plant have a greater role than just as a physical carrier of these viruses.

Our finding that RNA viruses have a broad host range and are freely circulating in the pollinator community has important implications on export/import and movement of managed pollinators that may bring in new or more virulent strains of existing pathogens into the environment, with the potential for deeper impact on our agro-ecosystems and natural ecosystems. Further research is needed to study the impacts of these viruses on specific species of non-*Apis* hymenopteran pollinators and to determine if IAPV or other viruses are linked to additional pollinator decline. The present study, along with the recent lessons learned from dramatic honey bee losses, emphasizes the immediate need to promote honey bee health, encourage use of native pollinators, and focus on the disease dynamics of pollinator community as a whole. The role of diseases in overall pollinator decline demands additional attention.

## Materials and Methods

### Sample collection to determine the source of the RNA viruses in the stored pollen or bee bread

In a preliminary study to determine if pollen foragers themselves were responsible for the virus being found in their pollen loads, 12 pollen foragers were collected as they were entering a colony at the Hill Top apiary at Penn State in 2005. The pollen pellets or loads (mass of pollen grains collected by honey bees, bumble bees and many other bee species, in their pollen baskets or corbiculae on hind legs) were removed from the honey bees with one pollen pellet extracted for RNA and the other kept for pollen identification. Some of the foragers were directly assayed for viruses and eight were kept for 24 hrs at 34°C, 50% RH (Relative Humidity) with sugar syrup and water. The foragers were dissected into two body regions, head plus first thoracic segment (containing salivary glands) (H/T1) and the remainder of the body consisting of second and third thoracic segments and abdomen (T2,3/A).

In a more expanded study, 65 incoming honey bee pollen foragers were randomly collected from the landing board at the entrance of the five hives in two different locations (24 km apart) in Centre County, Pennsylvania during the summer of 2007. Bees were put individually into plastic tubes with pollen pellets still intact on their legs. Both apiaries were free from any CCD symptoms and all hives appeared to be normal and highly productive, with many individuals and with most cells in the brood nest filled with either larvae or pupae (indicative of a healthy colony and queen). During the same time period other non-*Apis* hymenopteran species were collected using sweep nets from flowering plants near these apiaries. Non-*Apis* hymenopterans were also collected near apiaries harboring IAPV-infected honey bees and with a known history of CCD in Pennsylvania and New York. Some *Polistes* wasps were collected from Illinois near Urbana, Illinois from the field as well as established colonies. All the specimens were photographed for identification and whenever possible one representative of each type was pinned for proper identification and kept as a voucher specimen. All samples were immediately put on dry ice and stored at −80°C in the laboratory until analysis. Sixty-five honey bee foragers, 68 pollen pellets (including 65 from honey bees and 3 from non-*Apis* hymenopterans) and 55 other non-*Apis* hymenopteran specimens were analyzed for RNA viruses. Pollen pellets were carefully removed from frozen specimens and stored in separate 1.5 ml centrifuge tubes. After removing pollen, hind legs of bees were discarded and the remaining body was washed thoroughly with distilled water to remove any pollen. Pollen pellet color was recorded and a subsample of pollen was mounted on a slide for identification as described below.

### Pollen identification

Pollen pellets were separated according to the color and microscopic slides were prepared by mounting unacetolyzed pollen in high viscosity silicone oil [Poly (dimethylsiloxane), 200® fluid, viscosity 30,000 cSt]. Slides were then observed and photographed under a ZEISS Axioskop compound microscope for morphological characters including overall shape and size of pollen grains, the number, shape and arrangement of wall apertures, and the structure and orientation of the exine surface [68], [69]. Source plants were identified by comparing with the reference collection of microscopic slides prepared directly from identified plants collected from the same region.

### Association of the viruses with pollen

To obtain information about the association of the virus and pollen, a pollen washing experiment was conducted following the procedure used by Aparicio et al. [70], with slight modifications. About 10 g of pollen pellets collected from honey bee hives with pollen traps, were mixed together. A 100 mg subsample was then suspended in 1 ml Trizol, vortexed for 30 sec. and centrifuged at 5000 rpm for 5 min. Supernatant was then removed and this whole procedure was repeated four times. The washed pollen was then homogenized with Geno/Grinder 2000 (SPEX SamplePrep LLC) at 1300 strokes/min. for 3 min in 1 ml Trizol. This whole washing process was also performed using 1 ml of phosphate saline-Tween polyvinylpyrrolodone buffer [1M Phosphate buffer saline (PBS), 0.05% Tween-20, 2% Polyvinylpyrrolodone] at pH 7.4, except that the fourth washing in this case was done with 1% SDS (Sodium dodecyl sulfate) to remove virus particles tightly bound to pollen grains. Viral RNA was extracted from all these samples using the extraction process discussed below. These samples were then analyzed for DWV, and BQCV using RT-PCR.

### Test of infectivity of virus in stored pollen and honey

In Fall 2005, frames of bee bread (stored pollen) and honey were collected from colonies previously determined to have DWV (both from symptoms and with RT-PCR). Multiple cells of bee bread or honey were sampled at random from both sides of each frame and RNA was extracted from groups of 2–3 cells. RT-PCR was performed for DWV, SBV, and KBV. Only DWV was detected in the majority of the cells both for frames of honey or bee bread. These frames were stored at ambient temperature over the winter (fluctuating from below −6°C to 32°C), with protection from pests. Additional frames were power-washed to remove all deposits, leaving some wax; these were designated as “clean” frames. The wax did not have DWV as tested by RT-PCR.

Six months later in Spring 2006, new packages were placed into new hive equipment in an isolated apiary (Rock Springs Apiary) that had no known feral or managed colonies of honey bees located within 8 km. The surrounding area was forest, meadow and farmland. After one week when the colonies had established and the marked queens had began to lay eggs, egg samples (N = 4 samples of 5 eggs each, or 20 eggs per colony) and worker attendants (N = 15) were collected for each colony and analyzed for DWV, BQCV and SBV. A total of twelve packages or colonies were found to have workers free of DWV, KBV, and SBV; and the queens were laying virus-free eggs. These packages were randomly divided into three treatments with four colonies each: Controls (fed artificial bee pollen and sugar syrup, given “clean” frames), DWV-Honey (fed a frame of honey contaminated with DWV and artificial bee pollen), or DWV- Bee Bread (fed a frame of bee-bread contaminated with DWV and sugar syrup). Egg samples from each colony (N = 4 samples of 5 eggs each, or 20 eggs per colony) were collected every week for five weeks following introduction of the frames of food; and DWV and SBV infections and actin were determined by RT-PCR. Each marked queen was observed in its colony during the experiment, ensuring that the same individual queens were being monitored for viral infection.

### Greenhouse experiments to test if IAPV can be transmitted between honey bees and bumble bees

In 2008, six commercial greenhouse bumble bee colonies (a queen and approx. 100 workers) were obtained each from two major commercial bumble bee rearing facilities; Koppert Biological Systems (Romulus, Michigan, US) and Biobest Biological Systems (Leamington, Ontario, Canada). Three honey bee colonies were established along with three bumble bee colonies (*Bombus impatiens*) in each of four rooms (16′×20′) in a greenhouse, with separate equipment used in each room. All colonies of honey bees and bumble bees were tested for IAPV, DWV, BQCV, SBV, ABPV, CBPV and KBV when introduced into the greenhouse. For each bumble bee colony, five workers were tested upon arrival; for honey bee colonies, 20 workers and 10 egg samples were tested. All bumble bee colonies were found to have DWV and BQCV upon arrival; no KBV, SBV, ABPV or CBPV was detected. The Koppert bumble bees had no IAPV; however, the Biobest bees had the eastern strain of IAPV upon arrival. For the 2008 experiment, we focused only on Koppert bumble bees. The honey bee colonies had DWV, BQCV, and low prevalence of KBV; no IAPV was found in the honey bee colonies.

The rooms were shaded with shade cloth (70% blockage) and kept at 70–90°C with elevated humidity provided by continuous swamp coolers. In each room bees were allowed to forage by providing common food sources outside the colonies; 50% sugar syrup in a common feeder; Megabee bee diet (Castle Dome Solutions, Yuma, Arizona) as a dry powder in an aluminum tray; and blooming pollinator-friendly plants (4–6 each of blue spirea (*Caryopteris clandonensis*), blanket flower (*Gaillardia aristata)*, goldenrod (*Solidago* sp.) and sedum (*Sedum telephium*)). Bumble bees also had access to their internal sugar feeders that came installed in the hives, except when the colonies were fed with virus solution in Petri dishes.

One room was designated as IAPV+ and the other IAPV−; sampling, feeding, and observations of the bees were done carefully using separate bee suits, gloves and other equipment to minimize contamination. In the IAPV+ room, the honey bee colonies were each fed inside the colony with 2 ml semi-purified IAPV solution (approximately 5-7×10^9^ viral genome equivalents or the amount found in approximately 2 bees) in 30 ml 50% sugar syrup in a Petri dish; the IAPV- honey bee colonies were each fed inside the colony, 30 ml 50% sugar syrup in a Petri dish. Bees in each honey bee colony consumed 30 ml of sugar solution containing IAPV within few hours. Virus solution was prepared by crushing IAPV-infected honey bees in 1 ml/bee PBS (Phosphate buffer saline) buffer. Homogenate was then centrifuged at 5000 rpm for 3 min and passed through 0.2 µm NALGENE® syringe filters to remove any bacterial or fungal pathogens. From the inside of each colony, 30 honey bee workers and 5 bumble bee workers were collected every 3 days for first week and then weekly thereafter, frozen at −80°C, and assayed for IAPV using RT-PCR. Positive reactions were sequenced for confirmation.

This experiment was repeated in the summer of 2009, with commonly grown greenhouse vegetables and fruits. The plants included 35 strawberry (*Fragaria ananassa)*, 6 tomato (*Lycopersicon esculentum*), 8 cucumber (*Cucumis sativus*) and 2 blueberry (*Vaccinium angustifolium*) potted plants in each room along with other common ornamental flowering plants. Again, six bumble bee colonies (a queen and approx. 100 workers) were obtained from Koppert Biological Systems (Romulus, Michigan) and were kept with honey bees and same experimental procedures were applied. All six bumble bee colonies tested positive for the western strain of IAPV upon arrival. Three established honey bee colonies were split prior to use, with queens reared in each colony to emergence and mating before installation in the greenhouse as five-frame nucleus colonies. The control and virus-infected room each had one of the sister honey bee colonies. Each honey bee colony tested negative for IAPV prior to installation in the greenhouse.

### RNA extraction

Total RNA was extracted from individual samples using TRIzol® reagent (Invitrogen) and was resuspended in 20 µl DEPC-treated water. Concentration of total RNA was determined spectrometrically (Spectra Max 250, Molecular Devices).

### Reverse transcriptase-polymerase chain reaction (RT-PCR) for diagnostic and phylogenetic analysis

For diagnosis of the viruses, cDNA was synthesized using random hexamers and M-MLV reverse transcriptase (Promega), using the protocol of Cox-Foster et al. [16]. Primers were designed using Primer3 [71] except for BQCV primers that were obtained from Benjeddou et al. [72]. Different primer sets and their gene regions are listed in (Table S1). RT-PCR was carried out for DWV, IAPV, KBV and SBV using a program of initial denaturing for 8 min at 94°C and 35 cycles of 94°C for 55 s, 51.5°C for 55 s, and 72°C for 1 min 25 s, with a final extension step for 10 min at 72°C. For BQCV and ABPV, PCR was carried out using a program of initial denaturing for 8 min at 94°C and 38 cycles of 94°C for 1 min, 55°for 1 min and 72°C for 1 min 15 s, with a final extension step for 10 min at 72°C. Five micro liters of the RT-PCR products were electophoresed in a 1.5% agarose gel, stained with SYBR® Safe DNA gel stain (Invitrogen), and imaged using a Gel Doc XR (BIO-RAD). Primers (actin-F, ATGAAGATCCTTACAGAAAG; actin-R, TCTTGTTTAGAGATCCACAT) were used to amplify 514 bp of the honey bee actin gene (GenBank accession no. BI504901), serving as an internal control for the quality of RNA extraction. Detection of actin in the honey bees was used as a positive indicator of intact mRNA being assayed and also serves as an internal loading standard. A negative control lacking template DNA and a positive cDNA control were performed for each PCR reaction. Positive identification was confirmed by sequencing the PCR products. Primer sequences for DWV would have also amplified *Varroa destructor* virus-1 and Kakugo virus; sequence data did not find these viruses in our samples.

### Sequence analysis

PCR products were treated with ExoSAP-IT (USB) and sequenced on both strands. PCR products were also cloned using TOPO TA Cloning Kits with One Shot® Chemical Competent Cells (Invitrogen) and several individual clones were sequenced for each amplification. Sequence data were aligned and analyzed using the MEGA package. Nucleotide sequences determined in this study are deposited under GenBank accession numbers HQ655458-HQ655585.

### Phylogenetic analysis

Maximum likelihood (ML) phylogenetic trees were inferred for DWV, BQCV and IAPV. In all cases phylogenetic trees were estimated using the ML method implemented by the PAUP* 4.0 package [73] and utilizing the best-fit model of nucleotide substitution as determined by MODELTEST [74], which in each case was the most general time-reversible GTR+Γ_4_+I model (full parameter values available from the authors on request). A bootstrap resampling analysis of 1000 replications was performed to assess the support for specific nodes.

To statistically test if the evolutionary structure of BQCV, DWV and IAPV sequences is distinct among species, we computed the association index statistic (AI) [75] and parsimony score (PS) [76] statistic of clustering strength, using the BaTS (Bayesian tip-association significance testing) program developed by Parker et al. [77]. This analysis was based on a posterior distribution of phylogenetic trees inferred using the Bayesian Markov Chain Monte Carlo (MCMC) available in the BEAST package [78]. The BEAST analysis utilized the GTR+Γ_4_+I model of nucleotide substitution, a Bayesian skyline coalescent prior, and an uncorrelated lognormal relaxed molecular clock.

### Statistical Tests

SAS 9.1 statistical package (SAS Institute Inc.) was used to analyze the data by two-way ANOVA, Cochran-Mantel Haenszel statistical analysis, Fisher Exact test and Tukey-Kramer post hoc analysis. The BaTS program [77] was used to compute the association index statistic and parsimony score.

## Supporting Information

Figure S1 Color and number of pollen pellets having detectable virus. DWV = Deformed wing virus; SBV =  Sacbrood virus; BQCV = Black queen cell virus. N =  total number of pellets with detectable virus.
(1.70 MB TIF)

Table S1 Primer sequences for gene regions detected and sequenced for Israeli Acute Paralysis virus (IAPV), Deformed Wing virus (DWV), Kashmir Bee virus (KBV), Blackened Queen Cell virus (BQCV), and Sacbrood virus (SBV).
(0.06 MB PDF)
