# Supplementary figures and images for: RNA Viruses in Hymenopteran Pollinators: Evidence of Inter-Taxa Virus Transmission via Pollen and Potential Impact on Non-Apis Hymenopteran Species
Source: PLoS One. 2010 Dec 22;5(12):e14357. doi: 10.1371/journal.pone.0014357 (PMC3008715; doi:10.1371/journal.pone.0014357)

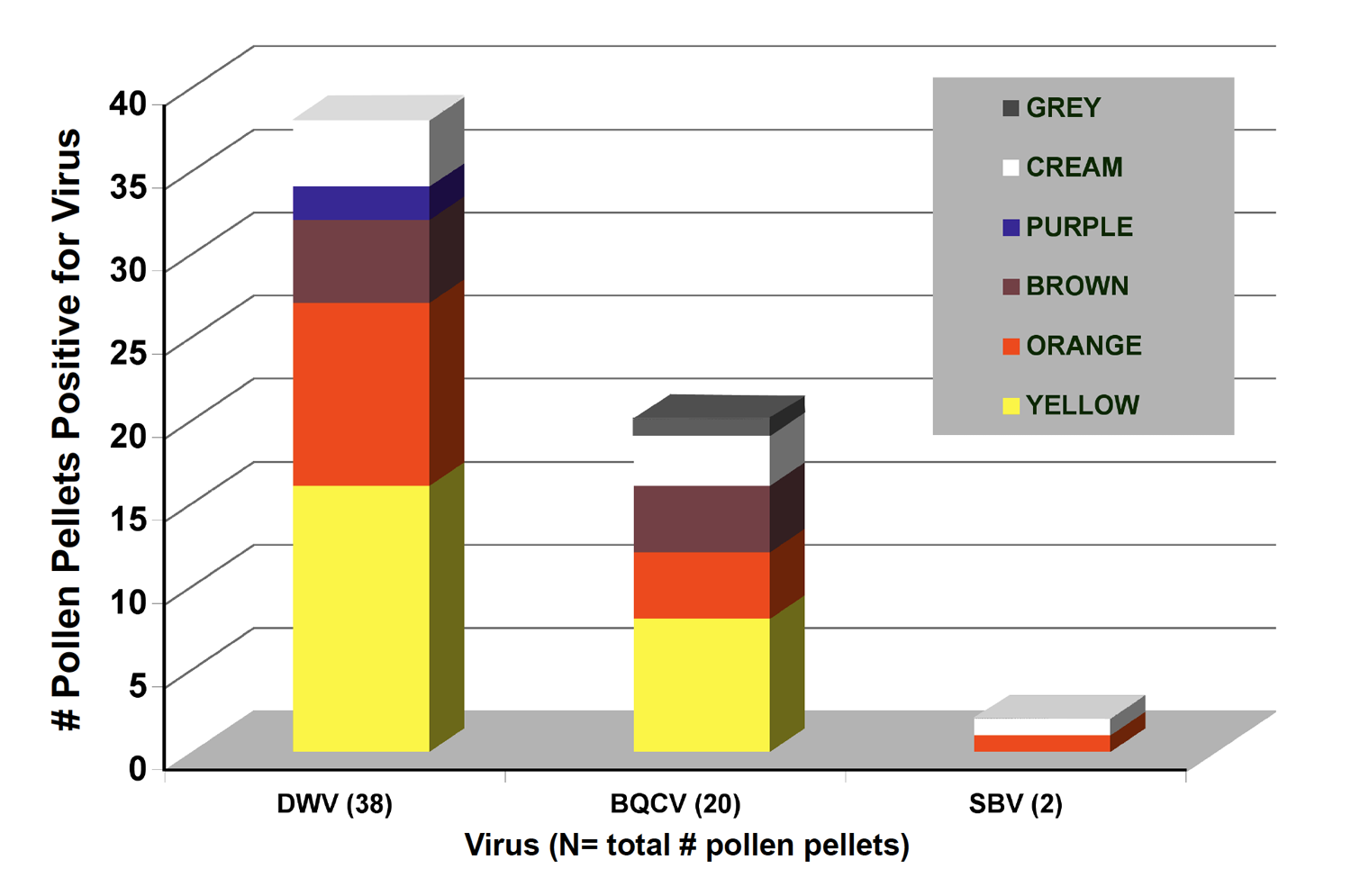

Supplement: Figure S1 — Color and number of pollen pellets having detectable virus. DWV = Deformed wing virus; SBV = Sacbrood virus; BQCV = Black queen cell virus. N = total number of pellets with detectable virus. (1.70 MB TIF) [file pone.0014357.s001.tif]
