# Supplementary material for: RNA Viruses in Hymenopteran Pollinators: Evidence of Inter-Taxa Virus Transmission via Pollen and Potential Impact on Non-Apis Hymenopteran Species
Source: PLoS One. 2010 Dec 22;5(12):e14357. doi: 10.1371/journal.pone.0014357 (PMC3008715; doi:10.1371/journal.pone.0014357)
Supplement: Table S1 — Primer sequences for gene regions detected and sequenced for Israeli Acute Paralysis virus (IAPV), Deformed Wing virus (DWV), Kashmir Bee virus (KBV), Blackened Queen Cell virus (BQCV), and Sacbrood virus (SBV). (0.06 MB PDF) [file pone.0014357.s002.pdf]

Table S1. Primer sequences for gene regions detected and sequenced for Israeli Acute Paralysis virus (IAPV), Deformed Wing virus (DWV), Kashmir Bee virus (KBV), Blackened Queen Cell virus (BQCV), and Sacbrood virus (SBV).

Virus	Primer Name	Gene	Location (nt)	Product (bp)	Accession No.	Forward (5'-3') Primer	Reverse (5'-3') Primer	Created by	Purpose or use in this paper
IAPV	IAPV Capsid1	Capsid	7776-8616	840	NC009025	GGTCCAAACCTCGAAATCAA	TTGGTCCGGATGTTAATGGT	This paper	Diagnostic assay or phylogenetic analysis
DWV	DWV VP1a	Capsid	2624-3047	424	NC004830	CTCGTCATTTTGTCCCGACT	TGCAAAGATGCTGTCAAACC	This paper	Diagnostic assay or phylogenetic analysis
DWV	DWV VP1b	Capsid	2948-3475	528	NC004830	GGCGTGGTTCATTAGAATATAGG	AAGCAGATCCCCACCTAAAAA	This paper	Phylogenetic analysis
DWV	DWV VP1c	Capsid	3415-3860	446	NC004830	GCTGGAGTGTGGCATAGCTT	CGAGACTCCTCTCCTTCTGG	This paper	Phylogenetic analysis
KBV	KBV Capsid1	Capsid	7941-8565	625	NC004807	TGTTTGTGGCAATCCAGCTA	TACGTCTTCTGCCCATTTCC	This paper	Diagnostic assay
BQCV	BQCV 3'UTR	Capsid/ 3'UTR	7850-8550	700	NC003784	TGGTCAGCTCCCACTACCTTAAAC	GCAACAAGAAGAAACGTAAACCAC	[56]	Diagnostic assay or phylogenetic analysis
SBV	SBV VP1b	Capsid	1655-2347	693	AF092924	GCACGTTTAATTGGGGATCA	CAGGTTGTCCCTTACCTCCA	This paper	Diagnostic assay or phylogenetic analysis
